# Detecting sarcopenia in obesity: emerging new approaches

**DOI:** 10.1097/MCO.0000000000001062

**Published:** 2024-07-18

**Authors:** Gianluca Gortan Cappellari, Michela Zanetti, Lorenzo Maria Donini, Rocco Barazzoni

**Affiliations:** a Department of Medical, Surgical and Health Sciences – University of Trieste; b Azienda Sanitaria Universitaria Giuliano Isontina (ASUGI), Trieste; c Department of Experimental Medicine, Sapienza University, Rome, Italy

**Keywords:** obesity, sarcopenia, sarcopenic obesity

## Abstract

**Purpose of review:**

Sarcopenic obesity is a likely common, but certainly underestimated obesity phenotype, with an important negative clinical impact. Its definition and diagnosis have however remained elusive until recently.

**Recent findings:**

Substantial progress has been recently made in sarcopenic obesity diagnostic tools, with the first international consensus proposed by the European Society for Clinical Nutrition and Metabolism (ESPEN) and the European Association for the Study of Obesity (EASO). Very encouraging results emerge from initial implementation of the ESPEN-EASO algorithm. In addition, even more recent progress in global consensus on sarcopenia conceptual definition is likely to further enhance consistency in sarcopenic obesity identification. The latter Global Leadership Initiative on Sarcopenia (GLIS) initiative also adopted a new definition of muscle specific strength. Its inclusion in sarcopenia diagnostic constructs opens the possibility of its potential evaluation in sarcopenic obesity, also considering the emerging positive impact of obesity treatment and fat loss on muscle functional parameters.

**Summary:**

New consensus tools for sarcopenic obesity diagnosis are likely to improve awareness, understanding, identification and treatment of this under-recognized obesity phenotype.

## INTRODUCTION

### Sarcopenic obesity: pathophysiology

Obesity is characterized by excess fat accumulation with negative health consequences [1,2], but it is increasingly clear that persons with obesity are at risk of more complex body composition derangements. Whereas high body mass may be associated with parallel increments of skeletal muscle mass in the general population [3^▪^,4^▪^], excess adipose tissue may per se be associated with muscle-catabolic derangements. Unhealthy nutritional habits with excess substrate availability and sedentary lifestyle may directly impair skeletal muscle protein anabolism [5^▪^], whereas primary metabolic alterations in expanding adipose tissue may lead to local and systemic oxidative stress, inflammation and insulin resistance, with direct muscle-catabolic impact [2,3^▪^,6^▪^,7^▪^]. Importantly, overweight and obesity are increasingly common in older adults, and older age is an important independent risk factor for loss of skeletal muscle mass and function [2,4^▪^,^8^]. It should be pointed out that both obesity *per se* and older age [3^▪^,4^▪^,5^▪^,^9^] are also major risk factors for a number of metabolic complications and systemic noncommunicable diseases (NCDs), as well as their acute complications. These may directly negatively affect muscle protein and energy metabolism, with negative synergistic impact on skeletal muscle mass, strength and endurance [3^▪^,7^▪^]. Finally, persons with obesity commonly undergo weight-loss treatments that inherently involve lean body mass and skeletal muscle [3^▪^]. Based on the above considerations, obesity with low skeletal muscle mass and function is likely a highly prevalent obesity phenotype, particularly in the growing population of older adults, and in patients living with comorbidities and NCDs at any age [3^▪^,4^▪^,5^▪^,^8^]. 

**Box 1:**
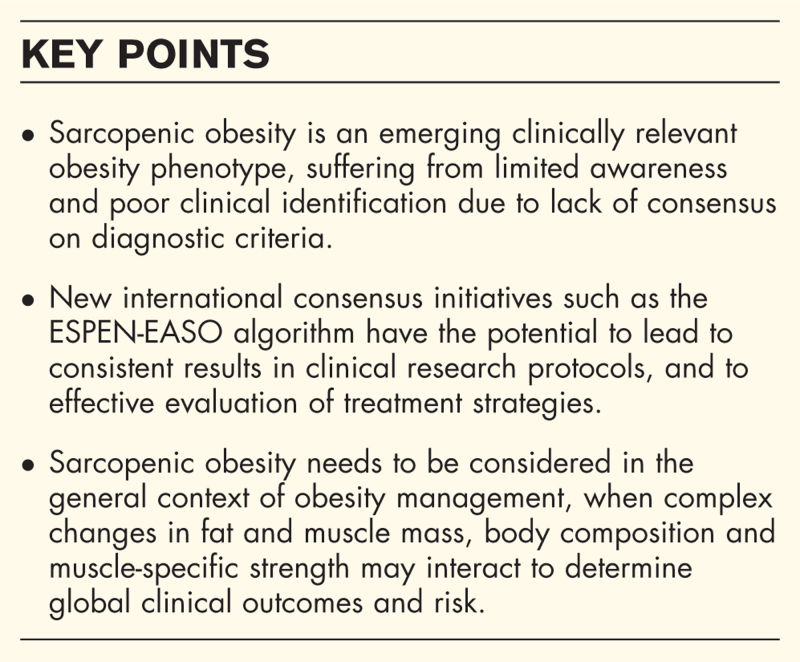
no caption available

### Sarcopenic obesity: clinical impact

The combination of obesity and low muscle mass and function, commonly defined as sarcopenic obesity, is associated with substantial negative impact on clinical outcomes [3^▪^,7^▪^,^10^–^13^]. Loss of muscle mass may negatively impact locomotor function and autonomy, but also whole-body glucose utilization, cardiorespiratory fitness and immune function [14]. Obesity with low skeletal muscle mass has been accordingly reported to be a risk factor for frailty, type 2 diabetes, impaired fitness [3^▪^], and in general with a higher risk of comorbidities and reduced survival in different settings [15,16^▪^]. A negative impact of obesity with low muscle mass has been specifically demonstrated in aging and in several disease conditions. In older adults, negative outcomes have been reported including cognitive impairment, loss of autonomy and mortality [12,17^▪^,^18^]. Obesity with low muscle mass or function is conversely associated with poor outcomes in different age groups in cancer, chronic heart failure, chronic kidney disease and chronic liver disease [8,13,19]. In the light of its likely increasing prevalence and its strong negative clinical impact, identification of sarcopenic obesity in clinical practice and implementation of effective prevention and treatment strategies are urgent clinical priorities.

### Sarcopenic obesity diagnosis: a difficult task and recent advances – the ESPEN-EASO algorithm

**FIGURE 1:**
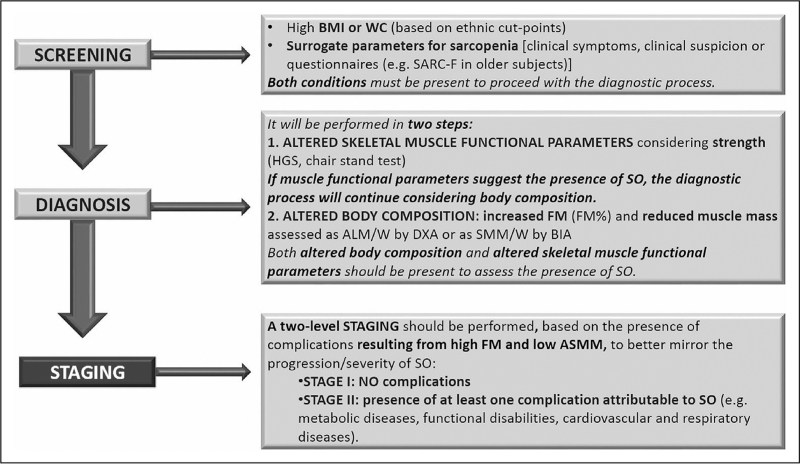
Diagnostic procedure for the assessment of sarcopenic obesity according to the ESPEN-EASO algorithm. ALM/W, appendicular lean mass adjusted to body weight; BIA, bioelectrical Impedance analysis; DXA, dual x-ray absorptiometry; FM, fat mass; HGS, handgrip strength; SARC-F, strength, assistance with walking, rising from a chair, climbing stairs and falls SMM/W, total skeletal muscle mass adjusted by weight; SO, sarcopenic obesity; WC, waist circumference. Previously published by Donini *et al.*[5^▪^].

Sarcopenic obesity has been until recently only defined and detected in clinical research through existing definition and tools for sarcopenia or obesity, in the absence of a unifying pathophysiological or clinical approach [19,20]. Importantly, ongoing disagreements and discrepancies in sarcopenia diagnosis have also impaired ability to uniformly define and diagnose sarcopenic obesity [4^▪^,5^▪^], resulting in lack of consistency and large variability in sarcopenic obesity prevalence in various clinical settings [19]. In recent years, however, initiatives to reach consensus definitions have been successfully implemented for both conditions. In 2017, the European Society for Clinical Nutrition and Metabolism (ESPEN) and the European Association for the Study of Obesity (EASO) have launched a call to action to tackle this issue [21]. A working group of international experts performed an initial systematic review to confirm whether common sarcopenic obesity definitions and diagnostic approaches had emerged in published articles [19]. The article clearly confirmed that no prevalent or preferred approaches were detectable – to the contrary, a number of criteria and combinations with variable cut-offs had been employed, expectedly confirming low consistency and comparability among results [19]. The group then proceeded to propose a consensus-based algorithm for sarcopenic obesity diagnosis, based on screening, diagnosis and staging steps (Table 1 and Fig. 1) [5^▪^]. The approach also aims at implementation in routine clinical practice, advocating for inclusive screening parameters and relatively simple methodologies for assessment of skeletal muscle function and body composition [5^▪^]. The ESPEN-EASO algorithm notably proposed a stepwise approach as indicated by most accepted sarcopenia diagnostic algorithm [22,23]. Also similar to the EWGSOP algorithm, the ESPEN-EASO diagnostic assessment dictates a preliminary measurement of skeletal muscle strength, and low muscle strength is needed to proceed to measure body composition [5^▪^]. Notable differences from the EWGSOP construct include the obvious inclusion of high BMI or waist circumference as a necessary component of sarcopenic obesity screening, and the need of both low skeletal muscle mass and high fat mass for sarcopenic obesity diagnosis [5^▪^]. Importantly, at variance with EWGSOP, the ESPEN-EASO algorithm dictates that skeletal muscle mass be normalized for total body weight, introducing the important concept that absolute muscle mass within normal range may not imply body composition homeostasis in obesity, as higher muscle mass may be needed to balance excess fat, both functionally and metabolically [5^▪^]. Following sarcopenic obesity diagnosis, the algorithm further advocates a staging step, based on sarcopenic obesity associated complications potentially directly induced by low muscle mass or function, including impaired functional performance and impaired fitness or cardio-metabolic diseases [5^▪^]. The authors also discussed the need to consider potential methodological and clinical confounding factors including careful evaluation of edema and excess body fluids, and called for validation studies and follow-up discussions to hopefully reach evidence-based, optimized recommendations, based on comparable results from methodologically homogeneous protocols [5^▪^]. The ESPEN-EASO initiative has also continued after the first publication, with a follow-up paper summarizing directions for future refinement and research [24^▪▪^].

**Table 1:** Clinical symptoms or Suspicion Factors for the screening of sarcopenic obesity according to the ESPEN-EASO algorithm. Previously published by Donini L.M. *et al.*[5^▪^]

Age >70 years
Chronic Disease Diagnosis (e.g. inflammatory diseases and organ failure or chronic disease) including but not limited to:
- Chronic Heart Failure
- Chronic Kidney Disease (particularly renal replacement therapy)
- Chronic Bowel Failure or Dysfunction
- Chronic Liver Disease (particularly NASH and liver cirrhosis)
- Chronic Respiratory Disease
- Chronic Neurologic and neurodegenerative Diseases
- Chronic Cognitive impairment
- Depression
- Organ Transplantation
- Endocrine diseases (e.g. metabolic syndrome, diabetes mellitus, hypercortisolism, hypogonadism, and corticoid treatment)
- Osteoarthritis
- Cancer (especially but not limited to chemotherapy of breast or prostate cancer)
Recent acute disease/nutritional events:
- Recent hospitalization (particularly but not limited to COVID-19, ICU stay, surgery)
- Recent major surgery or trauma with/without complications
- Recent sustained immobilization or reduced mobility (e.g. trauma, fracture, orthopedic disease)
- Recent history of reduced food intake (e.g. < 50% for > 2 weeks)
- Recent weight loss (including diet-induced voluntary weight loss and
- weight cycling syndrome)
- Recent rapid increase of weight
- Long standing restrictive diets and bariatric surgery
History-complaint of:
- Repeated falls
- Weakness, exhaustion
- Fatigability
Perceived progressive movement limitations

ICU, Intensive Care Unit; NASH, Nonalcoholic Steatohepatitis.

The ESPEN-EASO algorithm has been implemented since publication to investigate sarcopenic obesity prevalence in relevant populations, and its impact on clinical outcomes. Interestingly, studies in community-dwelling older adults reported a rather consistent sarcopenic obesity prevalence of 10.4% [25^▪^], 9.6% [26^▪^], 8.7% (men) and 10.4% (women), respectively [27], 9.0% [28^▪^], 7.3% [29,30^▪^] when considering people with BMI-based, ethnic cut-point-specific overweight or obesity. Another community-based study of older adults reported a lower 4.5% prevalence [31]; however, the latter study reported sarcopenic obesity prevalence relative to the whole population, including nonoverweight and nonobese participants [31]. Indeed, this may become a relevant confounding factor when comparing cohorts with different obesity prevalence, and it seems therefore appropriate to routinely also express sarcopenic obesity prevalence in the reference obesity population. Importantly, ESPEN-EASO algorithm-based sarcopenic obesity diagnosis was reported to predict various clinical outcomes, including quality of life [29], falls and poor functional outcomes [26^▪^], disability, cognitive impairment, multimorbidity and polypharmacy [31] and mortality [30^▪^]. Also relevant, established risk factors for sarcopenic obesity including physical activity level [27] and metabolic syndrome [28^▪^] were confirmed to predict incidence of sarcopenic obesity using the ESPEN-EASO algorithm.

In cohorts of patients with chronic disease, the ESPEN-EASO algorithm detected higher prevalence of sarcopenic obesity, with 29% prevalence in patients with chronic respiratory disease [32], 31.9% in patients with advanced nonsmall cell lung cancer [33] and up to 40% in older patients with diagnosed frailty and cardio-metabolic syndrome [34]. Also important, sarcopenic obesity diagnosis was a predictor of mortality in cancer patients [33], of functional outcomes in asthma patients [32], and was associated with bone strength in frail individuals [34]. Regarding acute conditions, studies in Japanese patients used the ESPEN-EASO diagnostic criteria to investigate the prevalence and clinical associations of sarcopenic obesity in patients acutely recovering from stroke [35–37]. All studies reported low prevalence of sarcopenic obesity below 5% in the whole population, with a higher 7.9% when calculating prevalence in patients with BMI above 25 kg/m^2^[37]. The latter study also demonstrated clinical associations between sarcopenic obesity and activities of daily living and dysphagia [37]. Interestingly, one of the studies also reported substantially higher prevalence of sarcopenic obesity (>23%) when excluding the screening step, implying that only a portion of the patients underwent the diagnostic procedure assessing muscle strength and body composition [36]. This observation may warrant a comment on screening criteria, since based on the original ESPEN-EASO consensus paper all patients with obesity in these studies could have been screened as positive with indication for diagnostic assessment, due to acute disease and hospitalization [5^▪^]. Finally, two studies directly compared sarcopenic obesity prevalence based on the ESPEN-EASO criteria to that observed using other, nonobesity-specific approaches [26^▪^,^38^]. Intriguingly, in both studies sarcopenic obesity prevalence based on EWGSOP2 criteria was significantly lower than the one indicated by ESPEN-EASO, thereby supporting the important role of obesity-specific criteria.

### Global leadership initiative on sarcopenia: sarcopenia consensus definition and implications for sarcopenic obesity

As previously mentioned, although the concept of sarcopenia has been introduced almost 40 years ago in the geriatric setting, consensus on sarcopenia definition and diagnostic criteria has been incomplete, also hampering sarcopenia awareness in clinical practice, and partly contributing to inconsistent results for sarcopenic obesity studies [19]. A Global Leadership Initiative on Sarcopenia (GLIS) has been recently launched, including representatives of the major existing diagnostic frameworks and international scientific Societies worldwide in the geriatric, nutritional and research fields. The group published in 2022 a glossary paper intended to promote consistency in nomenclature and definitions of commonly used concepts in sarcopenia research and clinical practice [39^▪^]. Among several others, the paper redefined the existing concept of ‘muscle quality’, previously introduced by the EWGSOP-2 definition to indicate derangements in muscle composition that may impair muscle function, despite apparently preserved mass – for example, muscle fat infiltration or fibrosis that are commonly observed in obesity or aging per se [40^▪▪^]. The term muscle quality was replaced by ‘muscle specific strength’, defined as muscle strength divided by unit mass [40^▪▪^]. Following a Delphi methodology, the GLIS consortium has then published the first global consensus-based conceptual definition of sarcopenia [40^▪▪^]. The definition confirmed the inclusion of skeletal muscle mass and strength, and it indicated the need to include muscle specific strength, in order to take into account common changes in muscle composition with direct impact on muscle function [40^▪▪^]. Of note, the paper did not provide operational algorithms for assessment of the three defining parameters, which should be introduced by future GLIS articles. In addition, a description of sarcopenia outcomes was provided, including derangements and diseases directly caused by the core components [40^▪▪^]. Similar to the sarcopenic obesity algorithm, sarcopenia outcomes include impaired physical performance and consequent disabilities [40^▪▪^].

### Sarcopenic obesity in the context of obesity management: body composition and muscle-specific strength

Increasing consensus on conceptual and operational approaches for obesity-independent sarcopenia diagnosis are likely to contribute to enhance consistency in sarcopenic obesity research and clinical assessment [39^▪^,^40^^▪▪^]. However, as previously mentioned, tools conceived for sarcopenia diagnosis in the absence of obesity may have sub-optimal ability to detect sarcopenic obesity in obesity patients [41,42], when weight-normalization of muscle mass may become a key step of diagnostic procedures. On the other hand, the potential role of muscle specific strength in sarcopenic obesity could be considered for optimization of the sarcopenic obesity diagnostic construct, given the relevance of muscle composition derangements in obesity as well as aging. A detailed description of current available approaches to treat sarcopenic obesity is beyond the scope of the current review paper. In addition, as discussed above, lack of consistency in sarcopenic obesity definition has inevitably led to difficult interpretation of available sarcopenic obesity treatment studies. However, a concise commentary on this topic will be provided, particularly in the context of obesity management and in the light of recent consensus advances.

It should be pointed out that rigorous, randomized-controlled studies on sarcopenic obesity are less numerous than those on sarcopenia, and mostly performed in older adult cohorts. Similar to sarcopenia [43], resistance-based exercise and protein supplementation are the most studied and most consistently effective strategies to treat sarcopenic obesity, with more positive results from combination therapies [44,45]. One recent meta-analysis focused on retirement age of 50–70 years, as a potential window of opportunity to prevent age-related deleterious changes in body composition [16^▪^]. The paper confirmed exercise and protein supplements to be the preferred, more effective approaches [16^▪^]. In the context of sarcopenic obesity, higher complexity of outcomes also makes evaluation of results more complex; changes in muscle strength and mass do not always occur in parallel, and they need to be considered in the context of changes in body fat and composition [16^▪^,^44^,^45^]. It is conceivable that consensus initiatives to define and diagnose sarcopenic obesity [3^▪^,5^▪^,^24^^▪▪^] may improve consistency of results, which will however also require consistent and comparable treatment protocols and patient groups.

Voluntary weight loss during obesity management is an additional specific challenge in sarcopenic obesity. Loss of lean mass is an inevitable component of weight loss also during voluntary calorie restriction, even to some extent when muscle loss prevention strategies are implemented [46]. In practice, development of sarcopenic obesity as a potential side effect of obesity management should be considered as a relevant clinical risk in the light of its negative clinical impact, particularly in older adults and patients with comorbidities [5^▪^,^24^^▪▪^]. Careful risk assessment should be performed in high-risk patients before implementing weight-loss treatments, including screening and assessment for sarcopenic obesity, potentially using the ESPEN-EASO algorithm [5^▪^,^24^^▪▪^]. On the other hand, the benefits of fat loss should be weighed against the risk of losing muscle mass and function, potentially even in patients with sarcopenic obesity risk or with diagnosed sarcopenic obesity, for example, in those in stable clinical conditions and in the absence of acute catabolic complications or active pro-inflammatory phases of chronic comorbidities. In particular, the recent consensus definitions for sarcopenic obesity and sarcopenia provide frameworks to evaluate potential loss of skeletal muscle mass in the context of concomitant treatment-induced fat loss and related mechanical and metabolic benefits.

(1) MUSCLE MASS LOSS and BODY COMPOSITION CHANGES: Implications for sarcopenic obesity diagnosis. The ESPEN-EASO algorithm introduced muscle mass normalization for total body weight, making the ratio of muscle and fat loss during weight loss a key parameter for treatment monitoring. Although muscle mass loss should always be minimized, a larger reduction of fat mass could anyway improve the muscle-fat ratio, potentially to the extent of reversing a previous sarcopenic obesity diagnosis, or improving the sarcopenic obesity phenotype. Pharmacological obesity treatment with second-generation GLP1 agonists or double GLP1-GIP agonists has revolutionized obesity management in recent years, and related studies may provide interesting insight about therapeutic weight loss and body composition – notably independent of structured prevention strategies to minimize muscle loss (such as enhanced protein dietary intake or exercise training programmes), that were not included in the treatment protocols. The STEP-1 study with the GLP1 agonist semaglutide induced an average weight loss of 16% total body weight, and approximately one third was attributable to lean mass measured by DXA [47]. In the SURMOUNT-1 study of the double agonist tirzepatide, weight loss was even more profound reaching 22% at highest dose, with approximately one quarter as lean body mass [48]. At the same time, due to profound reduction in body fat, body composition improved with higher percentage lean mass [47,48]. It is therefore conceivable that potential sarcopenic obesity patients could have no longer met sarcopenic obesity diagnostic criteria after treatment, despite losing significant amounts of muscle mass [5^▪^,^24^^▪▪^].
(2) MUSCLE MASS LOSS AND MUSCLE SPECIFIC STRENGTH. The ESPEN-EASO algorithm did not include muscle specific strength, that is, muscle strength per unit muscle size, previously defined as muscle quality [39^▪^], as a component of the diagnostic algorithm. Importantly, although neither study reported direct measurement of muscle strength, physical function scores significantly improved in treatment groups despite muscle mass loss in the recent STEP-1 and SURMOUNT-1 studies [47,48]. One study [48] also reported that improvements in physical function were observed across all age groups, although average participants in this study were 44 years-old, and older participants were conceivably a relative minority. Improved functional parameters are also commonly reported following even more substantial weight loss with inevitable muscle loss in patients undergoing bariatric surgery [49]. These combined observations indirectly and intriguingly suggest that muscle specific strength could have improved following treatment despite loss of muscle mass [46, 47]. This hypothesis is directly supported by previous studies in patients with sarcopenic obesity undergoing weight loss through hypocaloric diet with concomitant resistance or aerobic exercise to promote muscle mass maintenance and function [50]. In these studies, significant loss of muscle mass was confirmed to accompany weight loss even in the presence of well controlled exercise training [6^▪^,^50^]. Muscle loss was however associated with unchanged or increased muscle strength in groups undergoing aerobic or resistance exercise, respectively, with parallel improvement of body functional parameters [2,50]. Most importantly, although no calculation was provided of muscle strength-to-mass ratio, changes in muscle strength could have notably potentially modified staging or reversed the diagnosis of sarcopenic obesity.

Mechanisms potentially enhancing muscle specific strength during successful obesity treatment could include fat loss-mediated improvement of muscle composition, with reduction of muscle fat deposition [7^▪^]. In addition, fat loss is expected to improve systemic and tissue metabolic derangement associated not only with muscle protein catabolism, but also with impaired mitochondrial function and energy metabolism [2,7^▪^]. In particular, improved ATP production could have obvious, direct positive impact on muscle function, including strength and endurance. Exercise training could also provide additional metabolic benefits through improved mitochondrial function and lower oxidative stress and inflammation, also potentially improving muscle strength and performance, at least partially independently of muscle mass. The above considerations introduce important clinical issues and suggest the consideration of muscle specific strength for future refinements of diagnostic algorithms.

## CONCLUSION

Sarcopenic obesity is a likely common but certainly underestimated obesity phenotype, with important negative clinical impact. In recent years, substantial progress has been made in sarcopenic obesity diagnostic tools, with the first international consensus proposed by the European Society for Clinical Nutrition and Metabolism (ESPEN) and the European Association for the Study of Obesity (EASO). Validation and potential refinement of the ESPEN-EASO are needed and are underway, with encouraging initial results. In addition, even more recent progress in global consensus on sarcopenia conceptual definition (GLIS initiative) is likely to further enhance consistency in sarcopenic obesity identification. The new definition of muscle specific strength and its inclusion in the GLIS sarcopenia diagnostic constructs opens the possibility of its potential evaluation in sarcopenic obesity, also considering the emerging positive impact of obesity treatment and fat loss on muscle functional parameters.

## Acknowledgements

*None.*

### Financial support and sponsorship

*None.*

### Conflicts of interest

*Rocco Barazzoni has participated to Advisory Boards for Nutricia Reasearch, Pfizer, Novo Nordisk, Eli-Lilly and Boehringer. The other authors have no conflicts to declare.*

## REFERENCES AND RECOMMENDED READING

Papers of particular interest, published within the annual period of review, have been highlighted as:

▪ of special interest
▪▪ of outstanding interest
